# Acoustic accelerometer transmitters and their growing relevance to aquatic science

**DOI:** 10.1186/s40462-023-00403-3

**Published:** 2023-07-27

**Authors:** Robert J. Lennox, Sindre H. Eldøy, Lotte S. Dahlmo, Jordan K. Matley, Knut Wiik Vollset

**Affiliations:** 1 Norwegian Institute for Nature Research, Trondheim, Høgskoleringen 9, 7034 Norway; 2 NORCE Norwegian Research Centre Laboratory for Freshwater Ecology and Inland Fisheries, Nygaardsgaten 112, 5008 Bergen, Norway; 3 Ocean Tracking Network, Dalhousie University, 1335 Oxford St, B3H 3Z1 Halifax, Canada; 4 NTNU Vitenskapsmuseet, Erling Skakkes gate 47B, 7012 Trondheim, Norway; 5 College of Science and Engineering, Flinders University, Adelaide, SA 5042 Australia

**Keywords:** Biologging, Sensor, ODBA, RMS, Metabolism, Fisheries management

## Abstract

There has recently been great interest in the use of accelerometers onboard electronic transmitters to characterise various aspects of the ecology of wild animals. We review use cases and outline how these tools can provide opportunities for studying activity and survival, exercise physiology of wild animals, the response to stressors, energy landscapes and conservation planning tools, and the means with which to identify behaviours remotely from transmitted data. Accelerometer transmitters typically send data summaries to receivers at fixed intervals after filtering out static acceleration and calculating root-mean square error or overall dynamic body action of 2- or 3-axis acceleration values (often at 5–12.5 Hz) from dynamic acceleration onboard the tag. Despite the popularity of these transmitters among aquatic ecologists, we note that there is wide variation in the sampling frequencies and windows used among studies that will potentially affect the ability to make comparisons in the future. Accelerometer transmitters will likely become increasingly popular tools for studying finer scale details about cryptic species that are difficult to recapture and hence not suitable for studies using data loggers. We anticipate that there will continue to be opportunities to adopt methods used for analysing data from loggers to datasets generated from acceleration transmitters, to generate new knowledge about the ecology of aquatic animals.

## Introduction

Animals move at multiple scales; these movements are a fundamental aspect of ecology and recording these movements has formed a key part of ecological inquiry [51]. Movement allows animals to exploit temporally dynamic resources and capitalise on heterogeneity in biotic and abiotic landscapes. However, movement is costly and there is increasing focus on how animals conserve and expend energy and the consequences for individuals and populations [10, 63, 77]. Movement is most readily measured at macro scales as animals transit landscapes, sometimes performing heroic migrations around the globe [20]. However, macro-scale movements are underlain by micro-scale changes in an individual’s position,these changes can be tracked using small accelerometers, devices that record the position in space in up to three dimensions at high frequency in units of gravitational force (*g*).

Measurements of animal acceleration have become an important tool in the ecologist’s toolbox [9, 77]. Accelerometers are sensors that measure position in three dimensional axes at a user-defined sampling frequency, typically > 1 Hz [13]. Acceleration is measured in units of *g*, which are equivalent to the force of gravity in air 9.81 m/s^2^, representing a combination of static and dynamic acceleration. Static acceleration is the angular incidence of the device relative to the gravitational field and the dynamic acceleration is the component of the measurement attributable to movement of the device [62]. The static acceleration provides information on the position of the tag in space relative to the animal and for triaxial accelerometer loggers is frequently applied to identify posture [62]. These sensors have been adopted to many applications including loggers that can help ensure the quality of blood samples transported within hospitals [24], measure jumping performance of volleyball players [5], and to measure changes in gravity on Mars [40]. Accelerometers have also played an incredibly important role in providing detailed information about animal posture, movement, activity, and behaviour in captivity and in the wild [9].

Accelerometers are now part of many standard biologging packages but must be recovered to offload the high dimensional data that are too large to be transmitted. The important data provided by accelerometers have also been adopted for use in transmitters, which integrate an accelerometer that makes measurements at fixed intervals, summarises the data onboard the tag, and transmits it as a data package to a receiver [56]. Most tags summarise the data within a sampling window and transmit a data summary [70], but others (i.e., Sonotronics, Tucson, USA) can be programmed to record and transmit the range of values from the three axes within a time interval [23]. Accelerometers integrated into transmitters have different usage than accelerometer loggers (Table 1) and parameterisation of the devices by the user can determine the utility of the data as well as their translatability for comparison in other studies (e.g. [28]. The aim of the paper is to discuss standardisation of accelerometer transmitter specifications and synthesize use cases for accelerometer transmitter data to users with a focus on wild animals, although these tags have also shown promise for monitoring welfare of captive individuals (e.g. [58].

**Table 1:** Accelerometer transmitters are distinguished from loggers by a few important features that should be considered by analysts in the study design phase

Feature	Accelerometer loggers	Accelerometer transmitters
Data product	Acceleration in units of *g*	Data summary, RMS or ODBA or range of values
Data recovery	Stored onboard the device	Transmitted to a receiver
Sampling rate	Typically 5–300 Hz	Typically 5–12.5 Hz
Sampling window	None, all recordings are logged and time-stamped	User specified
Transmission interval	None	User specified (usually 30–120 s), but must be greater than sampling window
Axiality	2 or 3 axes	2 or 3 axes
Static acceleration	Integrated in readings	Filtered out onboard the device
Device attachment	Usually mounted externally	Often implanted internally
Recovery of device	Recapture animal or use timed release mechanism	No recovery necessary
Usage	Typically rechargeable and reusable	Single use
Common auxiliary sensors	Temperature, depth, magnetic field	Temperature, depth

## Literature review of specifications

Programming of accelerometer transmitters can be customised in various ways by the manufacturer to suit the needs of investigators. Similarly, attachment of accelerometer transmitters to animals (e.g., internal or external) also varies. To explore the selection of such specifications, a review of ecology-based acoustic telemetry studies using accelerometer transmitters between 2010 and 2019 was conducted. Peer-reviewed acoustic telemetry articles were identified using the following search term in Web of Science™(v.5.34): “Acoustic telemetry” OR “Acoustic tracking” OR “Passive telemetry” OR “Acoustic transmit*” OR “Acoustic receiver*” OR “Acoustic tag*” OR “Ultrasonic tracking” OR “Ultrasonic telemetry” OR “Fish track*” (see Matley et al. [42] for more information). The methods of each article were inspected to identify the use of accelerometer transmitters. For those articles that used these transmitters, the following user specifications were extracted: sampling rate, sampling window, operation mode, pairing with other sensors, and attachment method. Fifty-three articles were identified using acoustic accelerometer transmitters while tracking different species of fish (38), elasmobranchs (10), sea snakes (2), lobster (1), crab (1), cuttlefish (1), and conch (1).

The sampling rate is the number of measurements drawn by the transmitter per second. Almost all studies that provided sampling rate measurements used either 5 Hz (19/33) or 10 Hz (8/33) as a sampling frequency for accelerometer transmitters (Table 2), which is relatively low resolution compared to many loggers (Table 1). Two studies used a sampling rate of 20 Hz, but that is still below the > 30 Hz rate suggested by Broell et al. [7] for behavioural classifications of animals. Finally, two related studies used transmitters sampling at 200 Hz for high resolution identification of feeding behaviour [28, 29]. Brownscombe et al. [13] reported Atlantic bonefish swim up 6 Hz (tail beat frequency), but found that correlations between accelerometer loggers and metabolic rate in Atlantic bonefish were more variable at sampling rates < 10 Hz. They suggested that the sampling should occur at at least twice the maximum tail beat frequency using loggers. Wilson et al. [78] did not observe tail beats > 4 Hz and concluded that 10 Hz was sufficient sampling freqency with a Vemco (Innovasea, Halifax, Canada) transmitter for estimating swimming speed of sockeye salmon. Therefore, for most fish, 10–12 Hz should be sufficient, particularly when investigators are interested in measuring acceleration in two dimensions (i.e., tail-beat frequency). Lower sampling rates (i.e., 5 Hz) appear to be common in acoustic transmitters to optimize battery life, but may be limited in only providing general activity estimates.

**Table 2:** Summary information from literature review of accelerometer specifications in ecology-based aquatic acoustic telemetry studies

Item	Summary
No. articles using accelerometers	53
No. of unique species	Fish: 38 Elasmobranchs: 10 Sea snakes: 2 Lobster: 1 Crab: 1 Cuttlefish: 1 Conch: 1
Sampling rate	No. article reported: 33 5 Hz (19) 10 Hz (8) 20 Hz (4) 200 Hz (2)
Sampling window	No. articles reported: 30 range: 0.25–180 s mean: 34 s median: 25 s
Operational mode	0-Axis (1) 2-Axis (4) 3-Axis (48)
Other sensors	Pressure (39) Temperature (2)
Attachment method	Internal (39) External (14)

Numbers in brackets represent the number of studies associated with the item row

**Fig. 1:**
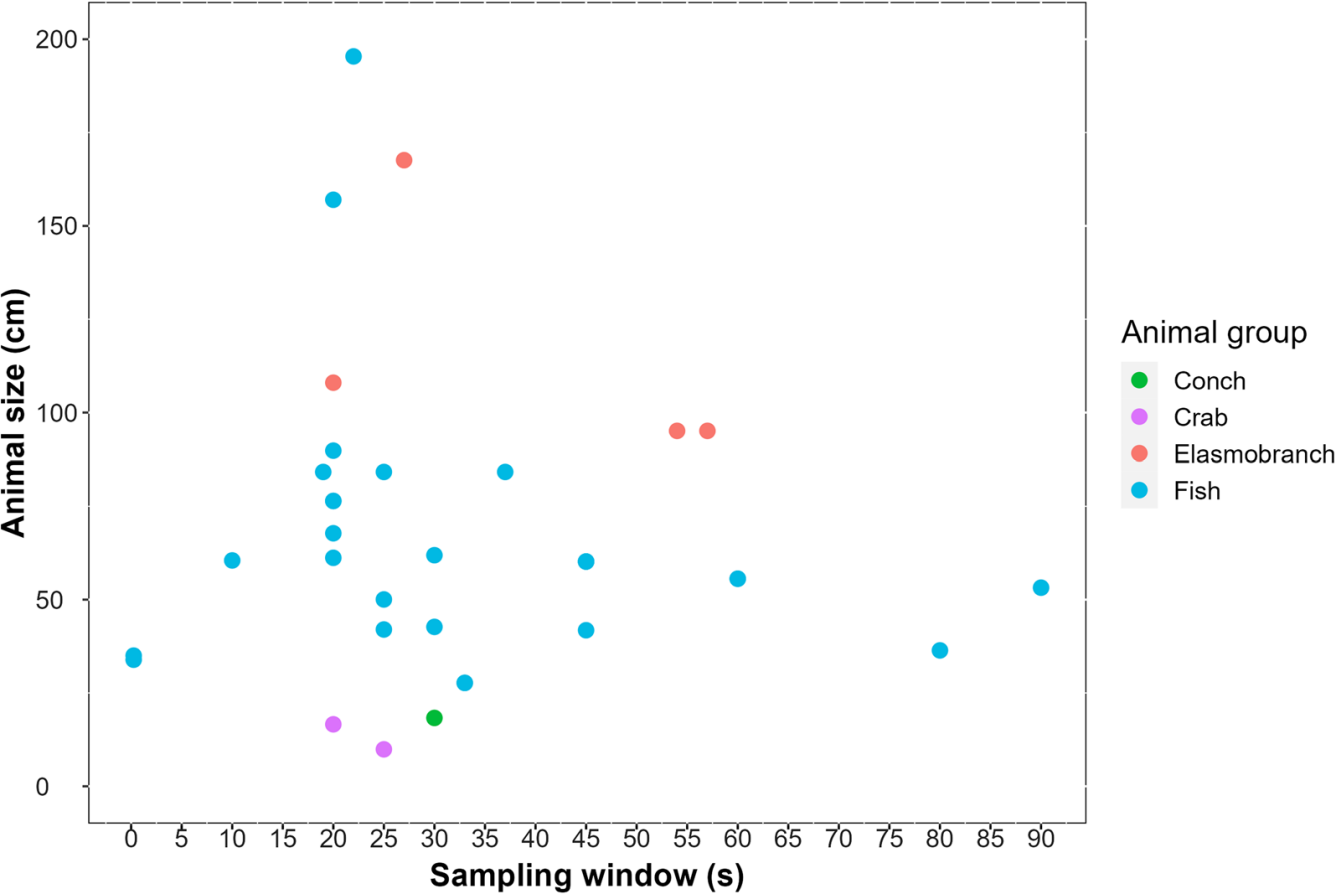
Distribution of sampling windows programmed into accelerometer transmitters in each study reviewed between 2010 and 2019 (30 out 53 studies provided this information). The colour of bar segments represent major taxonomic groups of the animals tagged. Elasmobranchs were categorised separate from fish for additional resolution

**Fig. 2:**
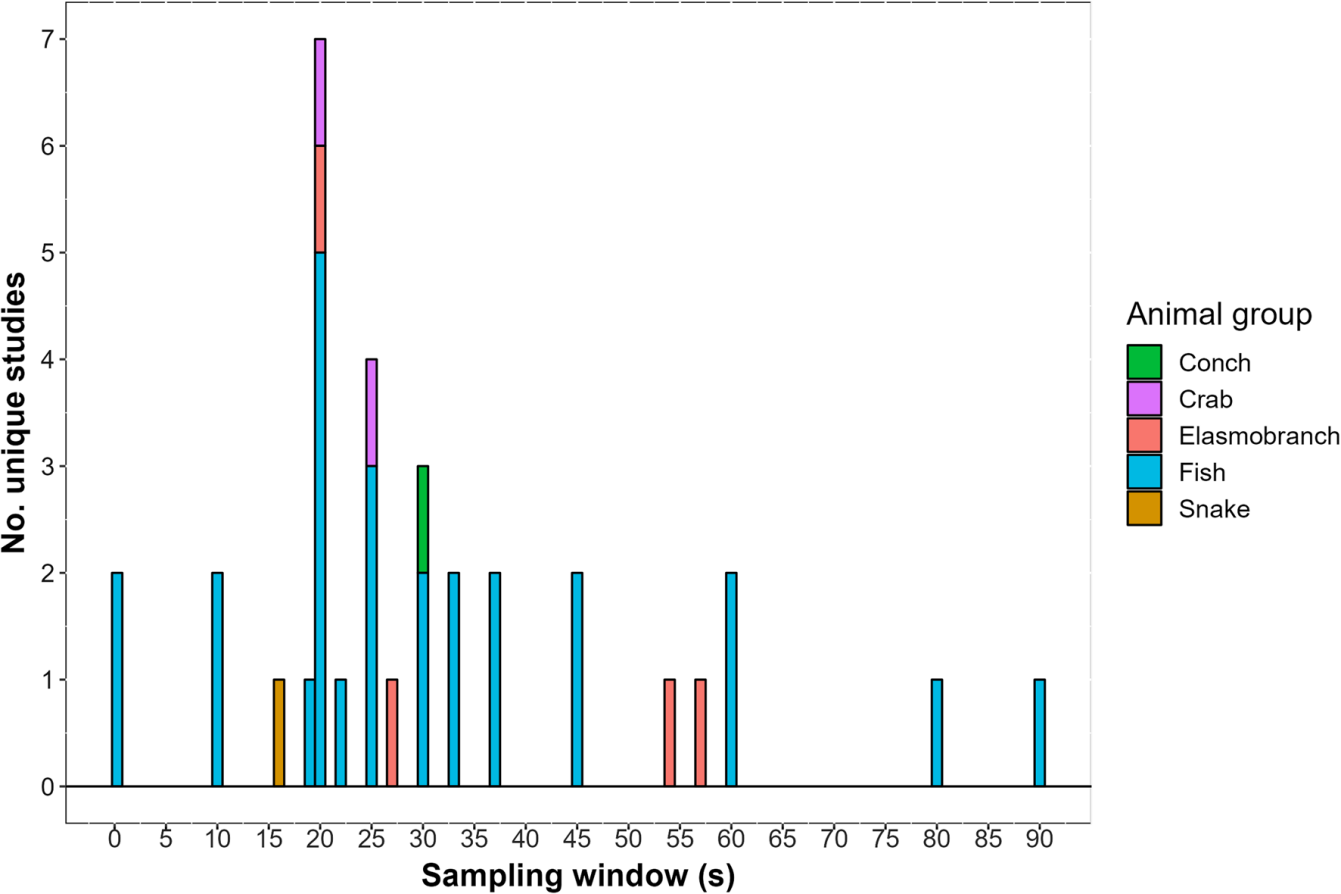
Relationship between sampling windows programmed into accelerometer transmitters in each study reviewed between 2010 and 2019 relative to the mean size of the animals tagged (30 out 53 studies provided this information). If more than one species was tagged in a study, they were each included. The colour of points represent major taxonomic groups of the animals tagged. Elasmobranchs were categorised separate from fish for additional resolution

The sampling window is the duration during which the measurements are drawn before they are summarised and transmitted. Shorter sampling windows will provide a more detailed snapshot of the activity at a given moment whereas longer sampling windows will yield a more synoptic view of the animal’s activity. Animals can shift their active states from sedentary to bursting at very short intervals so longer sampling windows are prone to include a more heterogeneous scope of the activity than shorter sampling windows. Moreover, shorter sampling windows better approximate the output of a high frequency tri-axial logger. The sampling window of studies reviewed ranged between 0.25 and 180 s, with mean and median values of 34 s and 25 s, respectively (Fig. 1). The sampling windows used was compared with the mean size of animals in each study to explore whether size-related trends existed in how users select this specification, but there was no clear pattern (Fig. 2).

Operation mode typically refers to one of two modes in which acceleration is estimated from either two or three axes. When two axes (roll and yaw) are measured, the purpose is to estimate acceleration from undulations of the animal’s body and not any motion up or down (pitch). Only a few studies (4/53) used this ‘tailbeat’ mode to measure caudal fin (i.e., fish) or mantle (i.e., cuttlefish) undulations (Table 2). The remainder used the ‘activity’ mode, which measures acceleration along all three axes (X, Y, Z) to provide a general estimate of activity. Notably, one study used an axis-free algorithm to measure feeding behaviour informed by a 3-axis acceleration tank experiment of red-spotted grouper (*Epinephelus akaara*) to reduce transmission burdens associated with the narrow bandwidth of ultrasonic transmissions [28].

Acoustic transmitters can also be constructed with different types of sensors in addition to (or instead of) accelerometers. These may provide additional information about the behaviour of the animal, such as whether it is moving (e.g., motion sensor) or measuring depth use (e.g., pressure sensor). Other sensors may also provide information about the surrounding environment (e.g., temperature and salinity sensors). Based on the reviewed literature, pressure (39/53 studies) and temperature (2/53) were the only additional types of sensors used (Table 2). The rarity with which temperature sensors were combined with accelerometers was surprising given that animal metabolism and activity scale with temperature, although temperature may be available from external data sources such as loggers integrated within receivers.

The final specification that was extracted from the literature review was the attachment method. The majority of studies (39/53) implanted acoustic transmitters internally, while the remainder attached transmitters externally (Table 2). Internal implantation almost exclusively incorporated inserting the transmitter through a small incision in the abdominal cavity and suturing the incision closed. Two of these studies sutured the transmitter to the inner wall of the abdominal cavity. External attachment followed various approaches with all studies (except one) securing firmly the transmitter to the animal's body to avoid motion not caused by the animal (e.g., water current). External attachment was performed on all invertebrates due to the hard outer surfaces providing adequate attachment areas, but elasmobranchs were also common recipients, for which the tag was attached to the dorsal fin. External attachment methods included epoxy, dart tag, clamp, saddle, and duct tape, among others. Whether internal or external, any implantation or attachment that leaves the transmitter moving independent of the animal is cause for concern and may affect both static and dynamic acceleration measurements [60]. Furthermore, the orientation of the transmitter should remain consistent and relevant to the goals of the study. When stated, all studies placed transmitters lengthwise in an anterior–posterior position to ensure acceleration forward and backward (Y axis) was represented appropriately. Still, tags may change orientation over time when implanted internally leading to biassed estimates until the body heals and encapsulates the transmitter in tissue. Therefore, secure attachment using multiple techniques, such as using additional sutures to secure transmitters to the internal body wall should be considered (but weighted against deleterious effects such as infection, incision size required to make the sutures, and surgery time).

## Applications

### Activity metrics and survival

Accelerometer transmitters provide a tool with which to observe activity of an animal within an array as well as infer survival. The activity transmitted by the accelerometers can be used to infer spatiotemporal dynamics, individual variation, or draw comparisons between sexes/among species, etc. Kadar et al. [32] used acoustic accelerometer transmitters to determine activity of Port Jackson sharks (*Heterodontus portusjacksoni*), demonstrating nocturnal peaks in activity and seasonal peaks indicative of migratory restlessness (i.e., zugunruhe). These transmitted data can be applied to understand environmental correlates of activity but can also be used to investigate energy partitioning and the limits of exercise for animals. Few investigations have attempted to determine the share of time partitioned into different activity states, for example calculating the time a fish spends at or near its maximum capacity for activity, but these transmitters provide a tool with which to observe activity budgets.

Natural mortality is a key component of animal demography but is challenging to establish in the wild [35]. Mortality of tagged animals can be determined from stationarity of tags or by depth profiles when tags are outfitted with depth sensors [36, 73]. Accelerometer transmitters can also provide high resolution information about the survival times of individuals in tagging studies. Unlike accelerometer loggers that are challenging to recover from dead animals, especially in aquatic environments, accelerometer transmitters yield data that can be accessed even from dead individuals that have otherwise disappeared (e.g. [18]. For several analyses, especially survival analysis (i.e. Cox proportional hazards), precise knowledge of survival time is required to accurately relate predictors to survival (Whoriskey et al. [75]). Accelerometer transmitters that reveal detailed individual histories are therefore key tools for investigating survival or modelling outcomes such as migration timing so that individuals can be appropriately censored from survival analyses.

### Exercise physiology (including calibrations)

Animals expend energy through aerobic and anaerobic pathways to accomplish diverse tasks, which can be linked both directly and indirectly to fitness [11, 12]. Acceleration transmitters can also be used to estimate the tagged animal’s exercise in terms of locomotion and energy consumption, as outlined by Cooke et al. [17]. Remote physiological data can be valuable for understanding the limits of sustained exercise for animals in the wild and their allocation of aerobic and anaerobic pathways on daily or seasonal bases. Transmitted acceleration data calibrated to respirometry have successfully provided new insights to swimming performance and energy expenditure of free-ranging aquatic animals, in many cases (but see [2]. For example, Payne et al. [53] estimated the swimming speed and oxygen consumption of giant Australian Cuttlefish (*Sepia apama*) aggregating at spawning grounds in the northern Spencer Gulf, South Australia, by calibrating transmitted acceleration data to observed swimming speeds and oxygen consumption. Combining acoustic telemetry and respirometry experiments have also provided detailed knowledge of the field metabolic rates of bonefish *Albula vulpes* [11, 12, 49, 52]. Wilson et al. [78] calibrated acceleration data to observed swimming speeds of tagged adult sockeye salmon *Oncorhynchus nerka* in a swim tunnel respirometer, and later used these calibrations to estimate swimming speeds and energetic costs of the migration in marine and riverine habitats [79, 80]. Bouyoucos et al. [6] demonstrated that semi-captive juvenile lemon sharks (*Negaprion brevirostris*) in a mesocosm had highest swimming velocities and energy expenditure during diurnal periods and flooding tides; these experimental approaches are effective for better understanding costs of life for animals in their environment. Some variation can be expected depending on the body plan and swimming mode of the fish, and Arechavala-Lopez et al. [2] did not find that accelerometer transmitters effectively identified states of sustained high activity swimming in sea bream (*Sparus aurata*).

Calibration experiments are useful for enabling comparisons between studies by transforming sensor output to common units such as swimming speed and energy expenditure, or by establishing direct relationships between output from different sensor types, tagging methods, or accelerometer-derived metrics. Using loggers rather than transmitters, Thiem et al. [71] linked four activity metrics (tail-beat frequency (TBF), tail-beat acceleration amplitude (TBAA), overall dynamic body acceleration (ODBA), and vectorial dynamic body acceleration (VeDBA) to the swimming speed of lake sturgeon *Acipenser fulvescens*, showing that all these metrics were good indicators of swimming speed for this species and how these metrics relate to each other; similar calibrations are possible with transmitters. Moreover, calibration studies make it possible to evaluate how well various acceleration metrics perform in distinguishing between the behavioural states of interest and is hence useful for pilot studies before launching large biotelemetry programs. ODBA and VeDBA were compared by Qasem et al. [57] who concluded ODBA was a slightly more reliable metric for correlating to oxygen consumption of humans and multiple captive birds and mammals. Bouyoucos et al. [6] found that tail beat frequency was a stronger predictor of metabolic rate than overall dynamic body action in juvenile Lemon sharks. Although most accelerometer transmitters use root mean square error as the summary function of the acceleration, it has not been as thoroughly evaluated from a physical or physiological perspective as has ODBA and a formal comparison would be beneficial.

### Response to, and recovery from, stressors

Stress is an adaptive response to cope with challenges that organism’s encounter, which often mobilises energy for a fight/flight response [81]. Responses to disturbance are likely to differ among species and may also have individual variation that has implications for the trajectory of populations based on the selective pressures placed on animals [4, 61]. Importantly, many natural stressors drive adaptive responses but for some anthropogenic stressors that scare animals but pose no threat to them, intensive stress responses may be maladaptive and drive selection for habituation that renders individuals more vulnerable to real stressors that they encounter [22]. Accelerometer transmitters have helped reveal the nature of aquatic animals responding to stressors, for example, Payne et al. [54] demonstrated that mulloway (*Argyrosomus japonicus*) cowered in response to boat noise and reduced activity during affected periods, a stress response with ecological implications for the species as well as conservation relevant applications for managing boat traffic. Moreover, van der Knaap et al. [72] used vectorial dynamic body acceleration of Atlantic cod (*Gadus morhua*) to exhibit a response to disturbances from seismic surveys.

Animals have primary, secondary, and tertiary responses to stressors and the metabolic costs of these responses must be repaid during a recovery period. Excess post-exercise oxygen consumption (EPOC) can be measured from animal respiration to quantify the costs of recovery from stress and determine the extent to which stressors impact individuals. The recovery period from stress is often characterised by low activity and distinguished from normal activity by changes in behavioural state. Moser et al. [47] demonstrated that accelerometer transmitters could be used both internally implanted and externally attached to identify breakpoints in acceleration indicative of recovery from gillnetting of green sturgeon (*Acipenser medirostris*). McLean et al. [43] used similar transmitters to determine that white sturgeon (*Acipenser transmontanus*) exhibited a brief period of swimming (10 min) following recreational capture before re-establishing a steady state of sedentary behaviour. Burnett et al. [14] is one of few to test hypotheses about fitness using accelerometer transmitters, demonstrating that gait transitions of sockeye salmon to anaerobic swimming at a challenging fish passage structure increased incidences of delayed mortality. Experiments that induce stress either in controlled or field contexts can evidently capitalise on the power of accelerometer transmitters to observe animal responses to challenge tests in the wild. However, approaches relying on these tools must be careful to control the intensity of the stressor in order to make useful inferences.

### Energetics and conservation planning

Energy is fundamental for movement, reproduction, and metabolism of any animal, consequently, availability of energy is critical for their survival [10, 77]. Lack of sufficient access to energy sources can greatly limit an animal’s trajectory, and maintaining an energy budget in which the expenditure of energy is minimised is essential for fish that are continuously exposed to the dynamic habitat that life in water provides [38]. Because energy expenditure can be estimated from acceleration data [83], use of accelerometer transmitters can provide a valuable tool to help reveal how aquatic animals partition activity and thus energy on spatial and temporal scales. Energetics data estimated from accelerometry can also be scaled across the lifetime of individuals to build individual-based models and estimate energy demands of the animal for growth, maturation, and the life course [65]. Green et al. [26] tracked invasive lionfish (*Pterois volitans* and *P. miles*) with acoustic tags implemented with acceleration sensors and revealed that they were more active during dusk and dawn and least active during night in Buck Island Reef National Monument, St. Croix, US Virgin Islands. This type of data can be valuable for successful culling of invasive species by documenting their temporal and spatial activity patterns. Based on tailbeat frequency, tail thrust, accelerometer and depth loggers, Tanaka et al. [68] suggested that the ascending phase performed by chum salmon (*Oncorhynchus keta*) was more energy demanding than the descending phase, illustrating that energy use might vary vertically in the water column, which could be much more tested using transmitters. Additionally, accelerometers can be combined with environmental data to reveal how activity is affected by abiotic variables and their transience through time (e.g., temperature, salinity, oxygen) as documented with an acoustically tagged Caribbean queen conch (*Lobatus gigas*, [66]. Environmental variables, such as prey availability, light conditions, or wave patterns, change temporally, and acceleration sensors can contribute to reveal how the behaviour of aquatic animals changes over time in response to changing environments. Lastly, the complexity of energy landscapes can be further expanded by combining depth sensors with accelerometers to create a three dimensional model of aquatic animal energetics. Modelling energy landscapes on horizontal, vertical, and temporal scales can advance the planning of aquatic habitats and protect habitats at multiple scales for a variety of species that not only exhibit horizontal and temporal movement, but often heavily rely on vertical movement to optimize their habitat exploitation. Regression models accounting for spatial dependency in activity or energy calculated from acceleration data have potential to improve the understanding of the spatiotemporal partitioning of activity exhibited by aquatic animals.

Energy landscapes, a term coined to describe the heterogeneity in costs of transport and other physiological expenses associated with habitat features [63], offer a synoptic view of the relationship between an animal and its environment that can be revealed by accelerometers. Fish often exhibit a varied preference of habitats over time (e.g., [21], and habitat selection might affect or be affected by energy demand and availability [27]. Thus, fish might exploit distinct areas at different times in which the allocation and expenditure of energy can be optimised via habitat modification. Shipley et al. [64] demonstrated with acoustic transmitters instrumented with acceleration sensors that the activity of Caribbean reef sharks (*Carcharhninus perezi*) were higher along the coral reef shelf southwest of Eleuthera, The Bahamas, and more evenly distributed along the shelf during night than during day. Furthermore, Kneebone et al. [37] used acceleration data from acoustically tagged juvenile sand tiger sharks (*Carcharias taurus*) in Massachusetts to visualise the spatial partitioning of activity by modelling Guassian Markov random fields using R-INLA [59]. Because accelerometer transmitters can help distinguish areas where animals exhibit higher activity (i.e., expends more energy) from areas where they exhibit lower activity (i.e., conserve more energy), combining tracking and sensor data can identify how habitats are utilised differently, for instance when foraging, resting, or seeking refuge [44, 69]. Identifying spatiotemporal energy landscapes and habitat function from transmitted acceleration data has great potential as an important tool for management and conservation, especially when boundaries are determined for marine protected areas (MPAs).

### Behaviour identification

Habitual and routine animal behaviours can be deconstructed into their component activities based on patterns in accelerometer data. Tri-axial accelerometers are able to be calibrated to identify behaviours using supervised machine learning algorithms such as random forest or support vector machines based on visual observations used to train the models (e.g. [16, 74]. One of the key constituents of behavioural identification for animals is using all three independent axes of the tri-axial accelerometer at high frequency [7], which is not available from data summaries sent by transmitters [28]. One-dimensional data from accelerometer transmitters are more challenging to use for behavioural identification but for species that have most movement in one plane (i.e., muscular contractions of the trunk and undulation of the caudal fin by fish), it may be easier. Karppinen and Erkinaro [33] mounted motion-sensitive radio tags that increased the pulse rate commensurate with the activity recorded by the tag below the dorsal fin of Atlantic salmon on spawning grounds and identified spawning behaviours of the salmon based on the activity metrics sent to receiver stations. Mulder et al. [48] stimulated forced swimming of Arctic charr (*Salvelinus alpinus*) in a field lab to identify acceleration values associated with resting, swimming, and bursting. Jury et al. [31] calibrated laboratory distances moved to transmitted accelerometer data and found a strong relationship such that field speeds could be calculated from a standard equation. Finally, Horie et al. [28, 29] demonstrated that an acoustic transmitter could even be pre-calibrated to transmit events identified by calibrations in the laboratory. Horie et al. [28] identified fish-eating and shrimp-eating behaviours by red-spotted grouper (*Epinephelus akaara*) using loggers, classified behaviours, and programmed a transmitter to identify and transmit the detected behaviours. Still, few seem to have attempted to match visual observations of aquatic animal behaviour to RMS or ODBA data products from transmitters for behaviour identification despite the broad application of this method in ecology using tri-axial loggers.

Unsupervised machine learning models may also be useful for behavioural disambiguation from acceleration data. Univariate time series of data products sent by transmitters (i.e. RMS, or ODBA) may provide the basis for Hidden Markov modelling (HMM) and unsupervised classification of behavioural states (Box 1). Leos-Barajas et al. [39] demonstrated how ODBA could be fit to these HMMs to terrestrial and aquatic time series. However, this has hardly been extended to data from transmitters. A key assumption when using Hidden Markov models for ecological data is that the data are recorded at fixed intervals,for acoustic transmitters that send data at a randomised delay, this means there must be an interpolation to generate a fixed series, which can be accomplished by linear interpolation or by model-fitting [30]. Moreover, it assumes that the animal does not exit the study system or have occluded detections that affect the time series. When HMMs are fit to these data, the user must specify the number of states for the algorithm to identify in the time series, typically 2–3 indicative of resting, moving, and perhaps bursting. Confined areas such as lakes or atolls where near-continuous time series of acceleration data are transmitted to receivers may lend to the use of HMMs for transmitted acceleration data whereas areas with incomplete coverage and patchy detection patterns will not be conducive. Runde et al. [60] fit HMMs to transmitted acceleration data from grouper in North Carolina to support fate classifications, and future users may find utility in their methods to discretize behavioural states from transmitted acceleration data.

**Box 1:** Example of accelerometer transmitter data from a sea-run brown trout (*Salmo trutta*) in a lake in western Norway covered by acoustic receivers

Accelerometer transmitter data for one brown trout (*Salmo trutta*) in a lake in western Norway are presented from September to December 2021. The trout were tagged with Thelma LP13-ADT transmitters with 27 s sampling intervals at 10 Hz in summer 2021 and this individual spent several months in the exorheic lake Vasbygdivatnet, where an array of 12 acoustic receivers recorded the transmissions in near-continuous time. The data series illustrated here comprises sixteen weeks of data through the spawning season of trout and into the overwintering period when the fish ostensibly conserve energy before returning to the ocean to recondition the following summer
This individual, 4718 (Fig. A), had an average acceleration of 0.17 m/s^2^ (Fig. B). The time series of acceleration was regularised to a fixed interval of 120 s using the *approx* function in R and a Hidden markov model was fit to the data to identify two discrete states using the *depmix* function in the depmixS4 package in R. The first state identified seemed to be slow, steady-state swimming whereas the second state seemed characterised by more rapid burst-type activity with higher sustained acceleration values. State transition probabilities were 17% from state 1 to state 2 and 4% between state 2 and state 1 (Figure C)
Positions of the individual in the lake were calculated using YAPS (Baktoft et al. [3]) following standard procedures to synchronise receiver clocks. A generalised additive model was fit to the acceleration data to examine the spatial effect using a gamma family and an interactive longitude, latitude smoother with k = 25. Model predictions illustrated relatively high predicted acceleration in the northwestern part of the lake, closest to the outlet that flows into the river Aurlandselvi. Predicted values also tended to be higher in the middle of the lake indicating the fish possibly used the centre of the lake to transit to the edges where it swam less vigorously

Simple data summaries and analytics derived from the accelerometer transmissions are presented

## Discussion

This paper addresses an increasingly used methodology in aquatic animal science and is intended to help investigators overcome common challenges that are encountered and perceived when designing studies with accelerometer transmitters and using the data. Accelerometer transmitters are different tools from their derivative, the tri-axial accelerometer biologger that is more readily used in terrestrial environments and for animals from which tags can easily be recovered. Although accelerometer loggers have been used for many aquatic species with pop-off packages, transmitters provide a much lower risk means of collecting activity or energetics data remotely given many aquatic animals are too small to carry pop-off packages or are too cryptic to be recaptured and recover the tag. Because accelerometer transmitters must have user-specified sampling windows and frequencies and transmit data summaries, the applications of accelerometer transmitters are somewhat different and users should consider these aspects as such when designing studies.

There is a need for better consideration in how accelerometer transmitters are specified by users. An overview of the literature indicated that most users are working with a sampling frequency of 5–10 Hz. Frequencies of 5 Hz may be too brief to effectively capture swimming dynamics of fast swimming species but 10 Hz is probably sufficient for most fish, albeit perhaps too infrequent for behavioural identification [7]. Apparently, the standard sampling frequency with Innovasea accelerometers is now 12.5 Hz, which was not captured in our review at all. Brownscombe et al. [13] suggested that biologgers should record at twice the maximum tailbeat frequency, which should effectively be covered by transmitters sampling at ≥10 Hz. Sampling windows in studies that we reviewed were much more variable, with devices ranging from 10 s windows to much longer intervals for summarising movement into RMS or ODBA. Shorter sampling windows (< 5 s) should be preferred for accelerometer transmitters to capture a more realistic snapshot of activity in a given window rather than averaging across a longer interval. Shorter windows are superior for capturing specific behaviours but mean that long periods of activity can be missed unless the transmission rates are adjusted to be frequent [70]. The use of code division multiple access (CDMA) tags rather than pulse position modulation (PPM) tags may allow for more frequent transmissions of higher frequency, shorter window acceleration measurements with lower risk of code collisions. Given the ubiquity of the Innovasea brand transmitters that use RMS as a data summary for transmission, this metric should be validated against ODBA and VeDBA to best understand performance of RMS as a metric. The best comparator is not clear, but Pereñíguez et al. [56] recently suggested that VeDBA is more resilient to variable sampling windows than is ODBA. ODBA has been validated as the best data summary from accelerometer loggers but VeDBA may be better when tags have the potential to move, as they do when implanted into fish without anchoring with sutures to the body wall [25, 57].

Acoustic transmitters are predominantly inserted into the intraperitoneal cavity of fish, but may also be attached externally to the dorsal fins of sharks, mounted on wires through the dorsal musculature of teleosts, glued to the carapace of turtles, or attached to claws of decapod crustaceans (as in Moland et al. [45]). The acceleration data obtained from tags attached or inserted using different methods will provide different results, but this has not been thoroughly tested. Attachment angles for accelerometer loggers have been investigated to develop best practices for accounting for individual variance in the placement of the tag relative to the body axis of the animal (e.g. sharks; [34]. Similar efforts would be justified to better understand how variation in tag insertion or attachment may affect metrics transmitted by the tags to receivers and for more species to guide users.

Our paper focuses on multi-axial accelerometer transmitters, however, there are other ways to use accelerometers that can add value to telemetry studies. It is possible to program tags with single axis accelerometer values to investigate, for example, animal pitch and transmit data about the movement toward or away from the water surface. Combining a tri- or bi-axial accelerometer transmitter with additional tag sensors can be valuable. Temperature measurements made by sensors and transmitted to receivers by the tag will represent either the external temperature experienced by the animal (for external attachments) or the internal temperature that adjusts to ambient water temperatures at a rate proportional to the animal’s size and the rate of temperature change [55]. Combining temperature and acceleration sensors on tags may provide the best possible resolution for relating individual activity to thermal biology compared to drawing inferences from nearby temperature logging stations (e.g. loggers embedded within the receiver) or models. Interestingly, we found that only a small number of published studies have combined these two sensors. Depth sensors combined with accelerometers may provide additional important context for the acceleration measurements transmitted by the tags, specifically the position in three dimensions of the animal when the activity is recorded. Dahlmo [19] combined depth and acceleration sensors in sea-run brown trout (*Salmo trutta*) in a lake and modelled the activity in horizontal and vertical dimensions to determine how the fish partitioned activity at different layers of the lake. In loggers, accelerometers are frequently coupled with magnetometers, which can add rotational information missed by accelerometers [76]. Magnetometer transmitters are not yet commercially available but may eventually provide additional layers of information to telemetry studies, but will require new calibrations and experimentation to better understand how sampling rates should be programmed and offset from alternating transmissions of summarised acceleration values for most efficient use.

We foresee new opportunities in the application of accelerometer transmitters for aquatic ecology. Our overview of key applications of these data to challenging questions in the aquatic realm suggests that several methods commonly applied to tri-axial accelerometer loggers are not well developed for accelerometer transmitters. Specifically, behavioural identifications using supervised and unsupervised machine learning methods may be promising for making use of acceleration data transmitted by aquatic animals; we predict that these tools will be more frequently applied and better integrated into aquatic research with accelerometer transmitters. Applying wavelet transform methods, commonly used to identify dominant spectra in logger data, may also contribute to identifying activity patterns and rhythms in data series from transmitters [15, 67],e.g. [50]. An important avenue for this research may include new data on biological rhythms including the nature of sleep in aquatic species based on cyclic periods of inactivity from transmitted acceleration data (e.g. [41, 82]. The ability to remotely infer sleeping in aquatic animals using transmitted acceleration data has the potential to open new research avenues into exercise physiology, ecology of stress, ecological chronotypes [1], and the nature of fitness (e.g. [14, 46]. In the future, we foresee new avenues for understanding aquatic animal exercise physiology including the limits of exhaustive exercise and the delayed effects of anaerobiosis and contrasts between natural and anthropogenic stressors [14]. Broell and Taggart [8] demonstrated potential for using acceleration metrics to track size at time (i.e. growth) of fish in the wild; this could have exciting applications for advancing ecology with transmitters as well as fisheries management, however, very specific calibrations would be needed to standardise placement of the transmitter and to program the accelerometer to identify the dominant tail beat amplitude. Eventually, combining data across studies applying these transmitters and using synthetic approaches to understanding how species’ activity levels are synchronised to their environment or even to each other could be possible. However, challenges will emerge in comparing transmitters that are programmed to sample at different frequencies and in different windows, are attached differently, and respond to scaling effects of the transmitter: animal body length differently. More empirical research would be justifiable in order to identify standard sampling frequencies and windows that can be more consistently applied for drawing comparisons across studies in the future.

## Data Availability

No data were used in the production of this manuscript.
